# Evaluation of Friction and Wear Behavior of Date Palm Fruit Syrup as an Environmentally Friendly Lubricant

**DOI:** 10.3390/ma12101589

**Published:** 2019-05-15

**Authors:** Mazin Tahir, Abdul Samad Mohammed, Umar Azam Muhammad

**Affiliations:** Department of Mechanical Engineering, King Fahd University of Petroleum and Mineral, Dhahran 31261, Saudi Arabia; s201264740@kfupm.edu.sa (M.T.); g201407600@kfupm.edu.sa (U.A.M.)

**Keywords:** date palm fruit syrup, environmentally friendly, tribology, friction, wear, FT-IR

## Abstract

The effect of various operational factors, such as sliding speed, normal load and temperature on the tribological properties of Date palm fruit syrup (DPFS) as an environmentally friendly lubricant, is investigated. Ball-on-disc wear tests are conducted on mild steel samples in the presence of DPFS as a lubricant under different conditions and the coefficient of friction and wear rate are measured. Scanning electron microscopy, stylus profilometry, and Fourier transform infrared spectroscopy are used to evaluate the wear tracks to determine the underlying wear mechanisms. Results showed that DPFS has excellent tribological properties in terms of low friction and low wear rates making it a potential candidate to be used as a lubricant in tribological applications.

## 1. Introduction

The value of the global market of lubricants reached USD 118.89 billion in 2016 and is further expected to grow over the next decades [1]. Lubricants are substances employed to undermine and regulate the induced wear and friction caused by the relative motion between different surfaces in contact. All lubricants consist of base oil, which serves as the elementary unit of a lubricant. Based upon the source of the base oil, lubricants can be categorized into mineral, synthetic or vegetable oils [2]. To further improve the properties of the base oil, it is mixed with modifiers or additives. Usually, additives are solid compounds dissolving in the base oil. They could be organic or inorganic in nature and they usually have a volume fraction ranging between 0.1 to 20 percent [3,4,5]. They assist with improving corrosion inhibition, viscosity index, and extreme pressure bearing ability and modifying friction [5]. Some of the most commonly used additives which have shown promising results in terms of improving the above properties are Zinc dialkyl dithiophosphate (ZDDP) and molybdenum dialkyl dithiocarbamate (MoDTC). However, it is to be noted that in spite of their excellent properties, these additives are a major source of air pollution which cause health hazards and contribute excessively to the global warming phenomena [6,7,8]. 

Hence, according to the World Energy Council, the energy sector will have to undergo transformations to overcome the global environmental concerns and to meet the global sustainable targets [9]. Thus, keeping in mind this transformation, a more environmental oriented tribology field called the ‘green tribology’ attained the researchers’ attention across the globe in the past few years. The green tribology field is more aligned with bio-degradability, recyclability and life cycle assessment [10] of green lubricants which utilize more natural base oil resources with more environmental-friendly additives. One of the more potential candidates for a green lubricant is the vegetable oil-based lubricants or fruit extract based oil lubricants. They are a promising alternative to typical mineral based lubricants due to their high lubricity, biodegradability and renewability [11,12,13,14,15]. Abdulqadir et al. [12] explored the feasibility of using various vegetable oils such as the red palm oil, sheabutter oil, black soap, ground nut oil and palm kernel oil for metal forming processes. They found that these vegetable oils showed lower coefficient of frictions as compared to that of dry conditions. However, the red palm oil showed the lowest coefficient of friction value of 0.039 among all the tested vegetable oils at room temperature. However, the shea butter oil outperformed the red palm oil at elevated temperatures in terms of lower coefficient of friction. Shankar et al. [13] evaluated the lubricating properties of kapok oil and found that it was very effective in reducing the friction and wear between two steel plates as compared to the palm oil and also a mineral oil (SAE 20W 40). Bahari et al. [14] evaluated the performance of two vegetable oils, namely, the palm oil and the soybean oil with the commercially available anti wear additive such as ZDDP. They found that the addition of ZDDP to soybean oil reduced the wear by 57% at a temperature of 100 °C.

Hence, the present study is a step in that direction whereby we are making an effort in exploring the feasibility of using syrup made of date palm fruit as a lubricant. Date palm (Phoenix dactylifera) is one of the most widely cultivated fruit in the Middle Eastern and some North African countries. Considering its excellent nutritional and medicinal values, it is extensively used in spreads, syrups and juice industries. Date palm fruit (DPF) has a rich agglomeration of nutrients, minerals and high levels of fatty acid groups. A comprehensive chemical study observed that DPF contains high amounts of Linoleic, palmitic and oleic acids [16]. Studies have shown that these fatty acids help in forming a thin low shear strength tribo-film because of the interaction between the carboxylic group which are mainly found in fatty acids and the metallic surface resulting in improved tribological performance [17,18].

To tap the presence of these fatty acids in DPF and foreseeing its potential application as a green lubricant, Samad [19], in his previous research, evaluated the effectiveness of date palm fruit syrup (DPFS) in reducing wear and friction under a constant load of 50 N and a sliding speed of 0.1 m/s. He found that DPFS showed excellent tribological properties in terms of reducing friction and wear and its performance was significantly comparable to that of an industrial lubricant (SAE 20W50). However, in his study, DPFS was evaluated at specific conditions of load (50 N) and speed (0.1 m/s) and temperature (room temperature = 23 °C). His findings are summarized in Table 1.

Therefore, the motivation of this study came from the fact that it is essential to explore the capability of DPFS to serve as a lubricant under wide operating conditions. Hence, in the present study, DPFS is evaluated under different normal loads, temperatures and sliding speeds to investigate its tribological performance for different potential applications. 

## 2. Experimental Details

### 2.1. Design of Experiments (DOE)

Due to the multivariable nature of the present study and in view of the inefficiency of one variable at a time technique which consumes a huge effort in terms of number of experiments especially when a wide range of data is considered and moreover, its inability to evaluate the interaction effect of different variables DOE and statistical optimization approaches such as Taguchi technique, response surface methodology (RSM), and factorial design are widely used to shift from the one-factor-at-a-time experimental approach. Taguchi methodology tries to find the optimized solution by employing fractional factorial design matrix. It can significantly minimize the total experimental effort by lowering the time and cost [20]. This technique is used to examine how different parameters with different combinations affect the mean and the variance of system outcomes [21]. Selecting appropriate experimental runs will reduce the number of experiments needed. For example, the Taguchi method helps to achieve the experimental results using fewer experimental runs and it offers a systematic approach to optimize the quality and performance [22]. The two major tools used in Taguchi design are the Orthogonal array (OA) and the signal-to-noise ratio (S/N ratio). The OA is a matrix of numbers arranged in specific rows and columns which should be selected properly from all possible combinations of the input variables/factors [23]. For example, L_9_ (3^3^) orthogonal design refers to an experimental design with nine runs, to study three factors, each at three levels. The signal-to-noise ratio (S/N) is the ratio of sensitivity to variability. The objective function of any system, in terms of the S/N ratio characteristics, may be categorized as; smaller is the best, nominal is the best, and larger is the best, respectively [22]. For our case, since the objective is to obtain low wear rate and low coefficient of friction, the smaller is the best characteristic of S/N ratio was considered, which is given as in Equation (1):

(1) $$\frac{S}{N}=10\;log\frac{1}{n}\left(\sum y^{2}\right)$$

where *n* is the number of observations, and *y* is the observed data. The factors and the levels used for each of the factors in the current research are listed in Table 2. 

**Figure 1:**
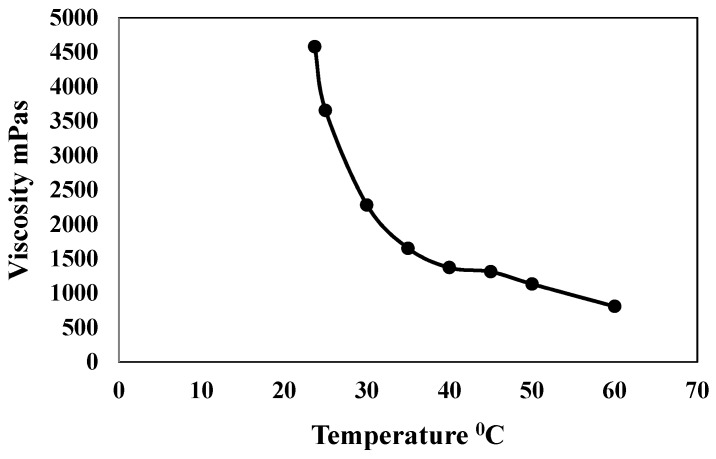
Variation of viscosity of DPFS with temperature.

The selection of the levels for the normal load and sliding speed were done with the aim of covering a wide range of operating conditions in most of the tribological applications. However, for the selection of the levels for temperature, we conducted a preliminary experiment to find out the effect of temperature on the viscosity of the DPFS as the viscosity plays an extremely important role in the tribological performance of any lubricant. The viscosity of the DPFS at different temperatures was measured by using a rotary type viscometer (Rotary viscosimeters “ST-2020”, JP Selecta, Barcelona, Spain). Figure 1 shows the variation of viscosity of DPFS with increasing temperature. It can be observed that the viscosity of DPFS decreased almost by 90% as the temperature increased from room temperature (25 °C) to 60 °C. Hence, we selected the levels for temperature to be from 25 to 60 °C to accommodate for the range of the viscosity drop.

### 2.2. Sample Preparation

Date palm fruit syrup bottles were bought from the local market for the experiments. Square mild steel samples of 25 mm × 25 mm × 5 mm were used as metallic substrates with a hardness of ~242 HV as measured using a MicroCombi tester (CSM instruments^®^, Zurich, Switzerland,) attached with a conical diamond tip of 2 μm diameter as an indenter. The coupons were uniformly ground and polished to an average surface roughness value of 0.1 ± 0.02 µm. The surface roughness was measured using the GTK-A Optical Profilometer (Bruker, Billerica, MA, USA).

### 2.3. Chemical Characterization of the DPFS Using Fourier-Transform Infrared Spectroscopy

Fourier-transform infrared spectroscopy (FT-IR, Bruker, Billerica, MA, USA) was used to conduct the chemical analysis of DFPS. FT-IR was conducted using Bruker’s VERTEX 70 FT-IR Spectrometer. Measurements were made at 50 scans and at a resolution of 2cm^−1^ in the wavenumber range of 4000 to 400 cm^−1^. Three repetitions were conducted and the average value is reported in the present study.

### 2.4. Wear Tests 

DPFS was used as a lubricant to conduct the wear tests on mild steel coupons using a ball-on-disc configuration on a Bruker UMT-3 tribometer (Bruker, Billerica, MA, USA) sliding against a hardened AISI 440C stainless steel ball of diameter 6.3 mm with a hardness of RC-62 and a roughness of ~0.35 µm. Different combinations of temperature (25, 40, 60) °C, speed (0.1, 0.2, 0.3) m/s and loads (50, 75, 100) N were investigated to see the contribution of each factor on the wear and the coefficient of friction. The wear test was conducted for 10,000 cycles corresponding to a sliding distance of ~185 m with a wear track radius of 2 mm. For every test, a sufficient measured quantity (around 45 mL) of DPFS lubricant was used to submerge the mild steel sample during the test. Table 3 shows the experimental design matrix (L_9_ OA) consisting of 9 runs with different combinations of the levels of each of the factors as specified by the Taguchi design methodology. Three replicates for each combination of the factors were performed, and the average value of the coefficient of friction and specific wear rates is reported. The coefficient of friction reported in the present study is the average dynamic/kinetic friction.

Optical microscope (Meiji, Tokyo, Japan) was used to evaluate the wear on the counterface ball by recording the images of the counterface ball before and after the wear tests. An Optical profilometer (GTK-A, Bruker, Billerica, MA, USA) was used to characterize the wear tracks by recording their 3D and 2D profiles after every test to calculate the specific wear rates (SWR) by using Equation (2):

(2) $$SWR=\frac{V}{N\cdot D}\cdot \frac{mm^{2}}{N\cdot m}$$

where *V* is the volume of the material removed which is estimated by multiplying the area of the 2D profile wear track as estimated by the Vision software (Vision64, Billerica, MA, USA) attached to the optical profilometer with the circumference of the wear track, *N* is the normal load in Newton, *D* is the sliding distance in meters. 

## 3. Results and Discussion

### 3.1. FT-IR Chemical Analysis of DPFS

**Figure 2:**
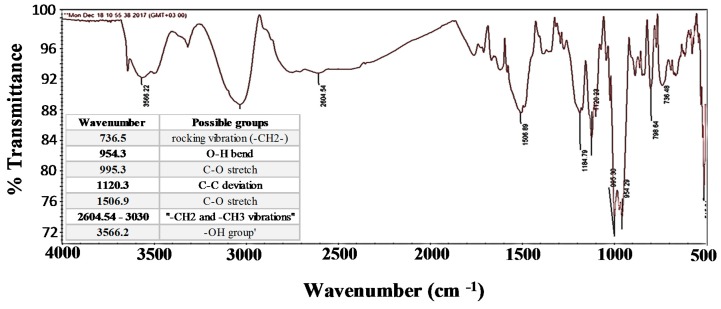
FT-IR transmittance spectra of DPFS.

The FT-IR spectrum of DPFS sample is displayed in Figure 2, and its respective peak readings are given in the inset table for clarity purposes. In Figure 2, the broad peak at 3566.2 cm^−1^ indicates the presence of a strong hydroxyl group [24,25]. The aliphatic −CH2 and −CH3 vibrations are seen between 2604.5 and 3030 cm^−1^ [26]. The peak at 1506.9 cm^−1^ is attributed to the stretching mode of (C=O) [26]. The peak at 1120.3 cm^−1^ is related to C−C deviation [27]. The bands at 900−1300 cm^−1^ are assigned to C−O bending modes of saccharides [28]. The peak at 954.3 cm^−1^ is attributed to the −OH group [29]. The peak at 736.5 shows a rocking vibration of (−CH2−) group [30]. All the above peaks are characteristic of different fatty acids such as the palmitic acid, oleic acid and linoleic acid which help in improving the anti-wear properties. 

### 3.2. Effect of the Input Variables/Factors on the Specific Wear Rate.

**Figure 3:**
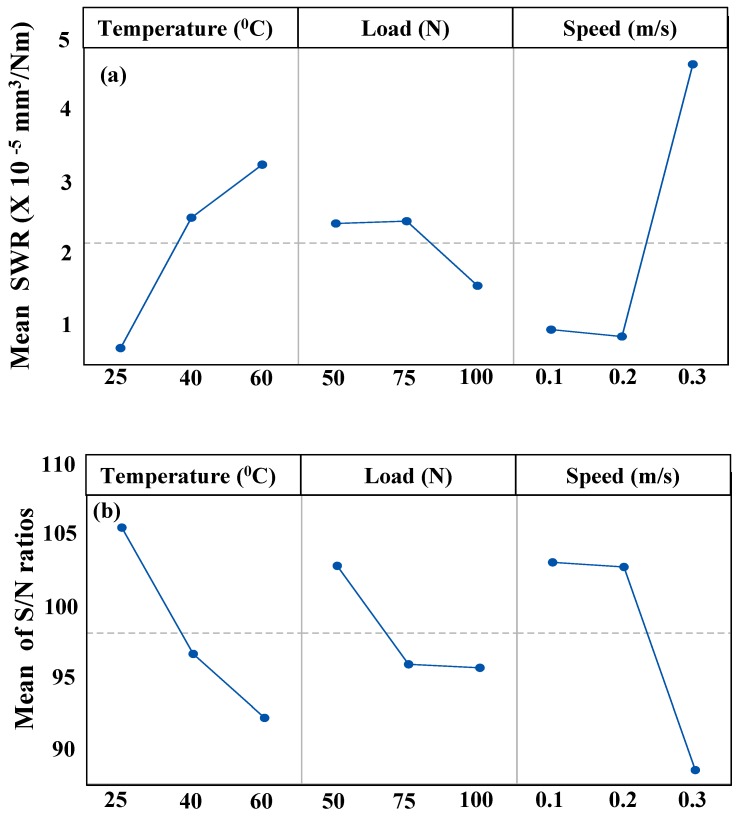
(**a**) main effects plot for the specific wear rate; (**b**) main effects plot for the mean S/N ratios, smaller is better.

Table 4 shows the response of specific wear rate (SWR) and the corresponding S/N ratio for the three trials of the experiment conducted at each of the combination of the levels of the three factors (Load, Speed and Temperature) for all of the nine runs. Figure 3a shows the main effects plots for the mean SWR for the different levels of each of the factors and 3b shows the main effects plots for the mean S/N ratios whose criteria in this case is ‘Smaller the better”. All these plots and the statistical analysis are done using Minitab 17 statistical software (Minitab, State College, Pennsylvania, USA). The plots are based on average values of each experimental run, and are used to evaluate the effect of each factor on the performance of DPFS. 

It is clear from the main effects plots that the specific wear rate increases with an increase in temperature from 25 to 60 °C, respectively. This can be attributed to the decrease in the viscosity of DPFS as mentioned earlier with an increase in temperature.

It is also observed from the main effects plots that the specific wear rate increases with increasing sliding speed. This can be attributed to the fact that with increased speed, the localized temperature at the point of contact increases which reduces the viscosity of DPFS leading to an increase in the specific wear rate. 

However, it can be observed from Figure 3a that with an increase in the normal load from 50 to 75 N, an insignificant change in the SWR was seen. However, as the normal load increased from 75 to 100 N, there was a significant drop in the SWR. This can be explained by two phenomenon taking place at the higher loads. Firstly, with an increase in the normal load, the asperities of the sample may have got plastically deformed resulting in a smoother surface which in turn contributes to the reduction in the SWR. Secondly, it can be attributed to the formation of an effective tribo film by the fatty acids present in the DPFS as evident from the FT-IR analysis which in turn helps in protecting the contacting surfaces at higher loads which simulates the boundary lubrication regime.

ANOVA analysis was also conducted at 95% confidence level (using a level of significance α = 0.05) to evaluate the contribution of each factor on SWR and are ranked as shown in Table 5. Based on this analysis, speed was ranked as the highest contributor to the specific wear rate followed by temperature and load as the second and third contributors, respectively. The optimal combination which will result in the lowest SWR comes out be 25 °C, 50 N and 0.1 m/s.

### 3.3. Effect of the Input Variables/Factors on the Coefficient of Friction

**Figure 4:**
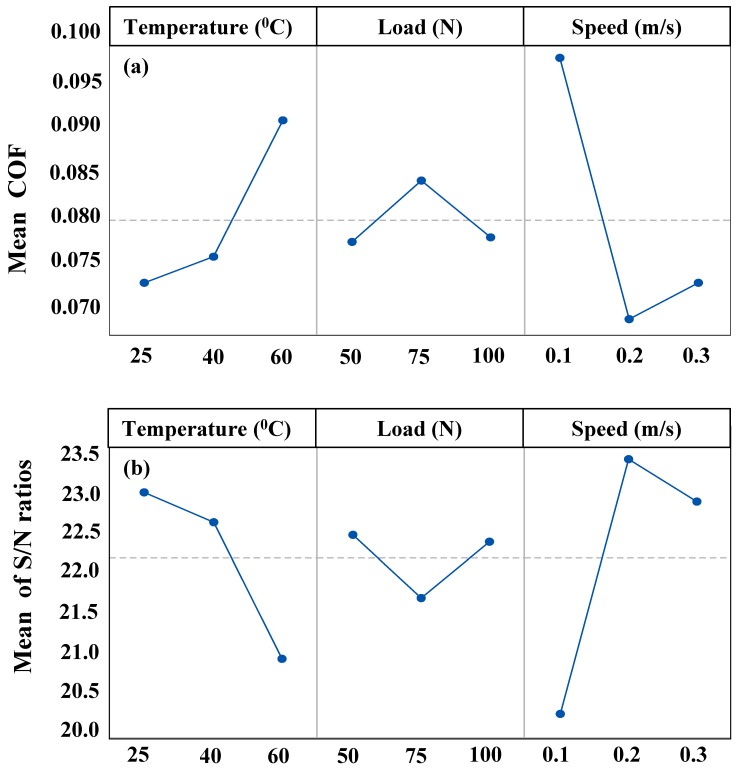
(**a**) main effects plot for the coefficient of friction; (**b**) main effects plot for the mean S/N ratios, smaller is better.

Table 6 shows the response of coefficient of friction (COF) and the corresponding S/N ratio for the three trials of the experiment conducted at each of the combination of the levels of the three factors (Load, Speed and Temperature) for all nine of the runs. Figure 4a shows the main effects plots for the mean COF for the different levels of each of the factors and 4b shows the main effects plots for the mean S/N ratios whose criteria in this case is ‘Smaller the better”. 

It can be observed from the main effects plots for mean COF for the different factors in Figure 3a that the mean COF increases with an increase in temperature. This can be attributed to the reduction in the viscosity of the DPFS solution with an increase in the temperature which may result in an intermittent metal to metal contact at the contact surface leading to an increase in the COF. 

However, with an increase in the normal load from 50 to 75 N, the COF seems to increase. This can be attributed to the fact that at lower contact loads, the DPFS separates the contacting surfaces with little or no asperities’ contact leading to a lower friction coefficient. To elaborate more, the real contact area increases proportionally with load (A_r_ = W/H, where A_r_ is the real area of contact, W is the applied normal load, H is the hardness of the softer material**)**, and frictional force increases proportionally to the contact load. However, with a further increase in the load to 100 N, the COF instead of increasing showed a significant decrease. This can be attributed to the extremely high load of 100 N due to which the contact region is deprived of the DPFS lubricant, which may have resulted in the plastic deformation of the asperities with the contact stress going beyond the yield stress of the material, thus resulting in a smoother surface leading to a lower COF and also lower wear rate as presented earlier.

It can also be observed from Figure 4a that the COF reduced drastically with an increase in the sliding speed from 0.1 to 0.2 m/s and showed a little increase with a further increase in the sliding speed to 0.3 m/s. The decrease in the COF can be attributed to the fact that at slow speeds hardly any lubricant is entrapped between the surfaces resulting in more metal to metal contact due to which the COF may be high, just as in the boundary lubrication regime. However, with an increase in the speed to 0.2 m/s, more and more DPFS lubricant will be pulled in the contact region, resulting in a reduction in the metal to metal contact leading to a reduction in the COF, just as in a mixed lubrication regime. However, the increase in the COF with a further increase in the sliding speed can be because of two reasons—first being that, as the speed increases more, DPFS lubricant will be pulled in the contact region separating the two surfaces completely, resulting in a slight increase in the COF just as in a hydrodynamic lubrication regime. However, it is to be noted that the SWR was higher at 0.3 m/s. Thus, the second and a more plausible explanation could be that, with an increase in speed, there may be a reduction in the viscosity of the DPFS lubricant due to an increase in the localized temperature, leading to a metal to metal contact resulting in an increased COF. 

**Figure 5:**
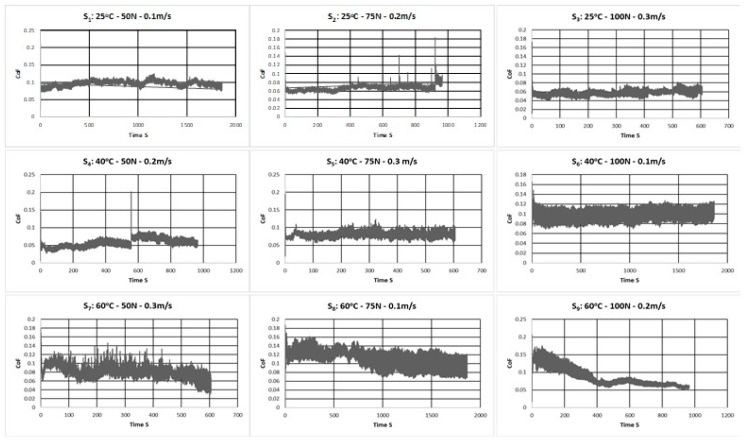
Typical frictional graphs corresponding to the different runs of the L_9_ array of the Taguchi Design.

Frictional graphs give a very detailed information about the various processes taking place during the test [31,32,33]. Figure 5 shows typical frictional graphs for the nine runs with the three factors running at different levels according to the L_9_ array of the Taguchi design. For most of the cases, the steady state COF is reached almost immediately after a very short running-in period with slight fluctuations. However, for the runs wherein the testing temperature was 60 °C and the load and the speed varied, more fluctuations in the COF graph are observed. This can be attributed to the reduction in viscosity of the DPFS lubricant. Moreover, higher loads simulate a boundary lubrication condition wherein the DPFS does not reach the contact region so as to protect the two mating surfaces. In particular, a higher initial COF is observed for the 9th run (60 °C, 100 N, 0.2 m/s) which could be due to the reduction in the viscosity of DPFS and also due to the higher load because of which a thick film of DPFS lubricant is not formed to separate the two mating surfaces. However, with time, the COF attains a steady state which can be attributed to the formation of a thin low shear strength tribo-film because of the interaction between the carboxylic group which are mainly found in fatty acids and the metallic surface, resulting in improved tribological performance of the DPFS.

ANOVA analysis was also conducted for the friction coefficient and the corresponding signal-to-noise ratio, resulting in the ranking of the factors which significantly affect the COF as shown in Table 7. The most dominant factor influencing the variation in COF was found to be the sliding speed. The least factor affecting the COF was the normal load.

The performance of the DPFS lubricant under the various conditions was compared to that of results of the sliding wear test of the hardened steel ball against the mild steel sample under dry conditions at a load of 50 N and a sliding speed of 0.1 m/s after 3500 cycles [19]. It is observed that the wear track depth with DPFS as a lubricant in all the experimental runs was much lower (~15 µm after 10,000 cycles) as compared to that of under dry conditions (~152 µm after only 3500 cycles) signifying the excellent performance of DPFS in protecting the mating surfaces from wear. Moreover, there was also a significant reduction in the COF from ~0.62 under dry conditions [19] to ~0.1 with DPFS as a lubricant highlighting the efficiency of DPFS lubricant in reducing both the COF and the wear rate.

### 3.4. Examination of the Wear Tracks and the Counterface Balls Using SEM and Optical Microscopy

**Figure 6:**
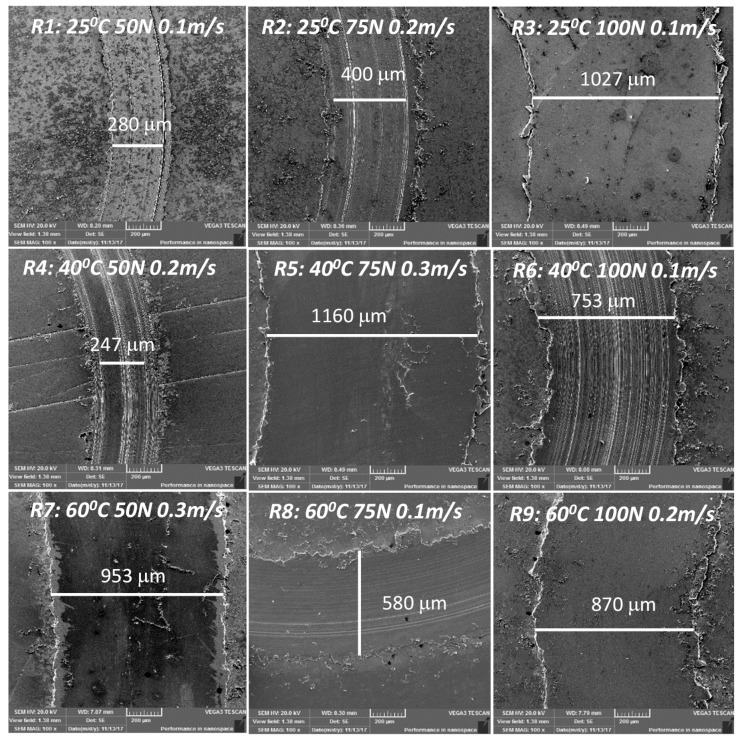
Scanning Electron Microscopy (SEM) images of the wear track after the test at different conditions at a magnification of 100×.

**Figure 7:**
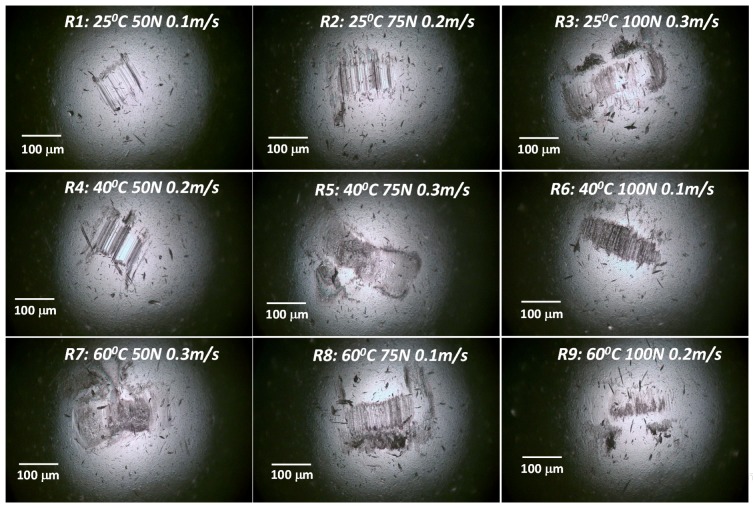
Optical images of the counterface ball after the wear tests conducted at different conditions according to the L_9_ OA.

On examining the SEM images of the wear tracks as shown Figure 6, it can be observed that both the wear track width in general increased with an increase in the load and sliding speed. However, on a closer examination of a few specific wear tracks such as that for R6 (40 °C, 100 N, 0.1 m/s), we found that the wear track was relatively rougher. This could be due to a possible metal-to-metal contact, resulting in the ploughing of the softer surface by the asperities of the hard counterface ball and resulting in a SWR of about 0.15 × 10^−6^ mm^3^/Nm. This could be due to the high load and low speed because of which there is not enough DPFS lubricant in the contact region, suggesting a boundary lubrication regime resulting in a metal to metal contact. However, the moderately low SWR value suggests the efficiency of the DPFS lubricant in protecting the two mating surfaces due to its ability to form a tribo film because of the presence of the fatty acids. Some of the wear tracks were also characterized by smooth surfaces, suggesting wear by plastic deformation as in the case of R9 (60 °C, 100 N, 0.2 m/s). This could be due to the flattening of the asperities in the first few cycles due to the high load and higher temperature whereby the viscosity of the DPFS lubricant reduces considerably. The R9 gave showed again a moderately low SWR of 0.171 × 10^−6^ mm^3^/Nm and a very low COF of 0.08. Moreover, a very small scar mark is observed on the counterface ball (Figure 7), suggesting the efficiency of the DPFS lubricant in protecting the mating surfaces.

It is also to be noted that the steel coupons showed a very low wear rate in general under the tested conditions as compared to the wear rates shown by few other vegetable/seed/fruit extract oils as found in the literature. Table 8 shows a comparison between the wear rates and the COF shown by DPFS and other vegetable oils under almost similar conditions. It can be seen that DPFS’s performance is quite comparable to that of the other oils in terms of wear rates and COF signifying its potential to be used as an environmentally friendly lubricant.

## 4. Conclusions

The focus of this study was to explore the capabilities of date palm fruit syrup (DPFS) under different operating conditions. Taguchi methodology of design of experiments was implemented to evaluate the effect of three factors, namely, normal load, sliding speed and temperature. Each of the factors was run at three levels to cover a good range of general operating conditions in tribological applications. Based upon the statistically designed experimental results obtained, it can be concluded that the date palm fruit syrup showed excellent properties in reducing the friction and the wear between the tribo-pair of mild steel coupons and a hard-stainless steel ball. DPFS was tested under various combinations of speed, load and temperature which reflect different mediums and applications. After running the experiment based on Taguchi matrix and doing ANOVA Analysis, sliding speed was found to be the most dominant factor in governing the specific wear rate (SWR) and the coefficient of friction (COF). It was also observed that, with an increase in the temperature, both the SWR and COF increased, which is attributed to the drop in the viscosity of DPFS. The normal load was found to be the least significant factor affecting the SWR and COF. 

## Figures and Tables

**Table 1:** Summary of the results obtained by Samad [19].

S. No	Lubricant	Coefficient of Friction (COF)	Depth of the Wear Track (µm)
1	Dry	0.62	152
2	Water	0.38	92
3	Industrial Lubricant (SAE 20W50)	0.09	11
4	DPFS	0.1	16

**Table 2:** Different factors and the selected levels for each factor.

Factors	Level 1	Level 2	Level 3
Normal load (N)	50	75	100
Sliding Speed (m/s)	0.1	0.2	0.3
Temperature (^0^C)	25	40	60

**Table 3:** The L_9_ Orthagonal Array showing the nine different runs with different combinations of the levels for each of the factors as specified by the Taguchi methodology.

Tests	Temperature (°C)	Normal Load (N)	Sliding Speed (m/s)
1	25	50	0.1
2	25	75	0.2
3	25	100	0.3
4	40	50	0.2
5	40	75	0.3
6	40	100	0.1
7	60	50	0.3
8	60	75	0.1
9	60	100	0.2

**Table 4:** Experimental results for SWR and the corresponding S/N ratios.

				SWR × 10^−6^ mm^3^/Nm				
Test #	Temp (°C)	Normal Load (N)	Sliding Speed (m/s)	Run # 1	Run # 2	Run # 3	Average	S/N Ratio (dB)
1	25	50	0.1	1.41	1.20	0.924	1.18	118.46
2	25	75	0.2	5.44	4.65	4.42	4.84	106.28
3	25	100	0.3	0.129	0.151	0.129	0.136	97.27
4	40	50	0.2	2.49	2.75	2.93	2.72	111.28
5	40	75	0.3	0.492	0.574	0.648	0.572	84.80
6	40	100	0.1	0.145	0.158	0.148	0.150	96.46
7	60	50	0.3	0.666	0.715	0.678	0.686	83.27
8	60	75	0.1	0.108	0.115	0.114	0.112	98.99
9	60	100	0.2	0.181	0.169	0.164	0.171	95.32

**Table 5:** Response table for mean SWR showing the ranking of the factors.

Level	Normal Load	Sliding Speed	Temperature
1	0.08	0.1	0.08
2	0.09	0.07	0.08
3	0.08	0.07	0.09
Delta	0.01	0.03	0.02
Rank	3	1	2

**Table 6:** Experimental results for COF and the corresponding S/N ratios.

				Coefficient of Friction (COF)				
Test #	Temp (°C)	Normal Load (N)	Sliding Speed (m/s)	Run # 1	Run # 2	Run # 3	Average	S/N Ratio (dB)
1	25	50	0.1	0.10	0.10	0.09	0.10	20.41
2	25	75	0.2	0.07	0.07	0.06	0.07	23.63
3	25	100	0.3	0.06	0.06	0.05	0.06	24.97
4	40	50	0.2	0.06	0.06	0.05	0.05	25.23
5	40	75	0.3	0.08	0.08	0.08	0.08	21.92
6	40	100	0.1	0.09	0.10	0.09	0.09	20.78
7	60	50	0.3	0.08	0.09	0.08	0.08	21.81
8	60	75	0.1	0.11	0.11	0.10	0.11	19.53
9	60	100	0.2	0.09	0.09	0.08	0.08	21.42

**Table 7:** Response table for mean SWR showing the ranking of the factors.

Level	Normal Load	Sliding Speed	Temperature
1	0.08	0.1	0.07
2	0.08	0.07	0.08
3	0.08	0.07	0.09
Delta	0.01	0.03	0.02
Rank	3	1	2

**Table 8:** Comparison of DPFS performance with a few other vegetable oils.

S.No	Lubricant/Oil	Specific Wear Rate (mm^3^/N.m)	COF
1	DPFS	1.74 × 10^−7^	0.08
2	Palm Oil [34]	3.97 × 10^−6^	0.4
3	Sunflower oil [35,36]	NA	0.05–0.15
4	Soybean oil [36]	NA	0.05
